# LostNet: A smart way for lost and find

**DOI:** 10.1371/journal.pone.0310998

**Published:** 2024-10-30

**Authors:** Meihua Zhou, Ivan Fung, Li Yang, Nan Wan, Keke Di, Tingting Wang

**Affiliations:** 1 School of Medical Information, Wannan Medical Collage, Wuhu City, China; 2 The University of Hong Kong, Hong Kong, China; 3 School of Physics and Electronic Information, Anhui Normal University, Wuhu, China; KUET: Khulna University of Engineering and Technology, BANGLADESH

## Abstract

The rapid population growth in urban areas has led to an increased frequency of lost and unclaimed items in public spaces such as public transportation, restaurants, and other venues. Services like Find My iPhone efficiently track lost electronic devices, but many valuable items remain unmonitored, resulting in delays in reclaiming lost and found items. This research presents a method to streamline the search process by comparing images of lost and recovered items provided by owners with photos taken when items are registered as lost and found. A photo matching network is proposed, integrating the transfer learning capabilities of MobileNetV2 with the Convolutional Block Attention Module (CBAM) and utilizing perceptual hashing algorithms for their simplicity and speed. An Internet framework based on the Spring Boot system supports the development of an online lost and found image identification system. The implementation achieves a testing accuracy of 96.8%, utilizing only 0.67 GFLOPs and 3.5M training parameters, thus enabling the recognition of images in real-world scenarios and operable on standard laptops.

## 1. Introduction

In urban areas worldwide, the confluence of increasing population density and the burgeoning volume of lost items poses a pressing challenge, exacerbated by the inefficiency of traditional manual search services. To address this exigency, the imperative for the rapid development of intelligent lost and found systems becomes manifest. Herein lies the genesis of our innovative approach—a fusion of multilingual prowess leveraging Python for deep learning model training and image processing, juxtaposed with Java for the construction of high-performance backend services enmeshing business logic and database interactions. This multilingual amalgamation not only augments system performance but also fortifies its maintainability.

Central to our innovation is the advent of the first-ever lost and found dataset meticulously curated from a gamut of sources including web crawlers, real-world photography, and structured surveys. Moreover, pioneering a paradigm shift in the lost and found domain, we introduce an avant-garde frontend-backend segregation strategy. Emboldened by an improved convolutional neural network (CNN) algorithm synergized with perceptual hashing, this stratagem liberates the user interface, image processing, and deep learning model inference from backend services. The resultant enhancement in system flexibility and maintainability is palpable, marking a watershed moment in lost and found system architecture.

Crowning our endeavor is the introduction of the LostNet algorithmic framework—a tour deforce amalgamating MobileNet V2, the CBAM attention mechanism, and the Perceptual Hash Algorithm. This integration heralds a new era in lost item image classification, delivering unparalleled performance while facilitating online recognition and matching services. In a grand synthesis of innovation and sustainability, our approach not only diminishes reliance on manual labor but also expeditiously and accurately discerns lost item categories, effectuating a paradigm shift in green development strategies while significantly curbing human service costs for transportation operators.

## 2. Research background

The utilization of convolutional neural networks (CNNs) spans various scientific domains, particularly in the realm of image recognition and classification [1, 2]. Academic attention has markedly shifted towards exploring and applying image recognition techniques. Within the domain of trash classification, a method for classifying garbage images was developed [3]. This method is based on an enhanced version of MobileNet V2 and incorporates transfer learning to enhance the real-time performance and accuracy of garbage image classification models. Yang et al. [4] attempting to identify plant leaves by the utilization of a hierarchical model that is based on CNN. A study on establishing the optimal size of the training data set that is required to achieve high classification accuracy with low variance in medical image classification systems is presented by Cho et al. [5]. Purnama et al. offer a method for the classification and diagnosis of skin diseases that are suitable for use in teledermatology [6].

It has also been demonstrated that transfer learning is beneficial in a variety of contexts. CNNs are used in the methodology that Lee et al. [7] propose as a fine-grained classification method for large-scale plankton databases. The implementation of transfer learning in CNN is one potential solution. Liu et al. [8] apply unsupervised transfer learning to CNN training to address these problems. Specifically, they transform similarity learning into deep ordinal classification with the assistance of several CNN experts who were pretrained over large-scale-labeled everyday image sets. These CNN experts jointly determine image similarities and provide pseudo labels for classification. Purwar et al. [9] make use of some models that are related to CNNs to identify mesangial hypercellularity in MEST-C. Herzog et al. [10] concentrate on the classification of MRI for the diagnosis of early and progressive dementia by utilizing transfer learning architectures that employ CNNs, as a base model, and fully connected layers of SoftMax functions or Support Vector Machines-SVMs. Phankokkruad [11] proposes the three CNN models for detecting lung cancer using VGG16, ResNet50, and DenseNet20 architectures. The proposed method is based on transfer learning.

CNNs have emerged as indispensable tools in image matching tasks due to their capacity to autonomously learn features from raw image data [12–14]. This ability enables CNNs to effectively represent and match features, making them essential for tasks requiring precise localization and recognition of visual elements [15–17]. Hierarchical semantic image matching methods leveraging CNN feature pyramids have emerged as a pivotal approach in image retrieval, aiming to enhance retrieval efficiency and precision [18]. These methods exploit the hierarchical representation of CNN features to bolster the accuracy of image retrieval processes. Additionally, in partial duplicate image detection, a novel strategy integrating Scale-Invariant Feature Transform (SIFT) and CNN feature matching has been proposed [19]. This hybrid approach amalgamates conventional feature extraction techniques with deep learning-based feature representations, enhancing detection performance by capitalizing on the complementary strengths of both methodologies. Moreover, advancements in cross-modal retrieval leverage CNN visual features to establish new performance benchmarks and devise sophisticated semantic matching techniques to tackle challenges inherent in cross-modal retrieval scenarios [20].

In summary, in image matching applications, CNNs play a pivotal role in extracting discriminative features from input images, which are subsequently compared and matched using diverse similarity metrics. This approach surpasses conventional methods reliant on manually engineered features or heuristic algorithms. CNNs’ scalability and adaptability render them versatile across a spectrum of image matching scenarios, ranging from fine-grained object recognition to wide-area surveillance and real-time video analysis.

## 3. Methodology

Smart lost and found solutions, such as BOUNTE (https://bounte.net/), utilize AI image recognition through a smartphone app to identify and log items, auto-tagging images with detailed descriptors and adding them to the venue’s digital lost and found system. However, despite its advanced features, BOUNTE is primarily designed for commercial use in settings such as hotels, limiting its broader applicability. Other solutions, such as blockchain-based platforms for decentralized management [21], university campus systems with enhanced security measures [22], location-based systems that integrate GIS functions for efficient tracking [23], and smartphone applications for managing lost items with contact features [24], also show significant advancements but are often context-specific. To address these limitations, a photo matching network is proposed that combines MobileNetv2 [25] with Convolutional Block Attention Module (CBAM) [26] Attention and the simplicity and speed of Perceptual Hash Algorithm [27] to efficiently compare images of lost and recovered items. A methodical approach is recommended by utilizing the improved MobileNet V2 and an intuitive graphical user interface (GUI).

To address the complex challenges associated with the image dataset of lost and found items, extensive research was conducted, including a sample survey among students at our institution. Questionnaires were distributed across various colleges, resulting in 2500 issued and 1986 valid responses. A private dataset was generated using web crawlers, real-world photography, and research examples. The most common lost and found items were categorized into 10 distinct groups. This study introduces a deep learning framework, termed LostNet, designed to facilitate the online recognition and matching of user-uploaded images of lost items. The model effectively addresses the labor and time costs inherent in traditional methods, providing an accurate and comprehensive solution backed by rigorous experimental data.

**Fig 1:**
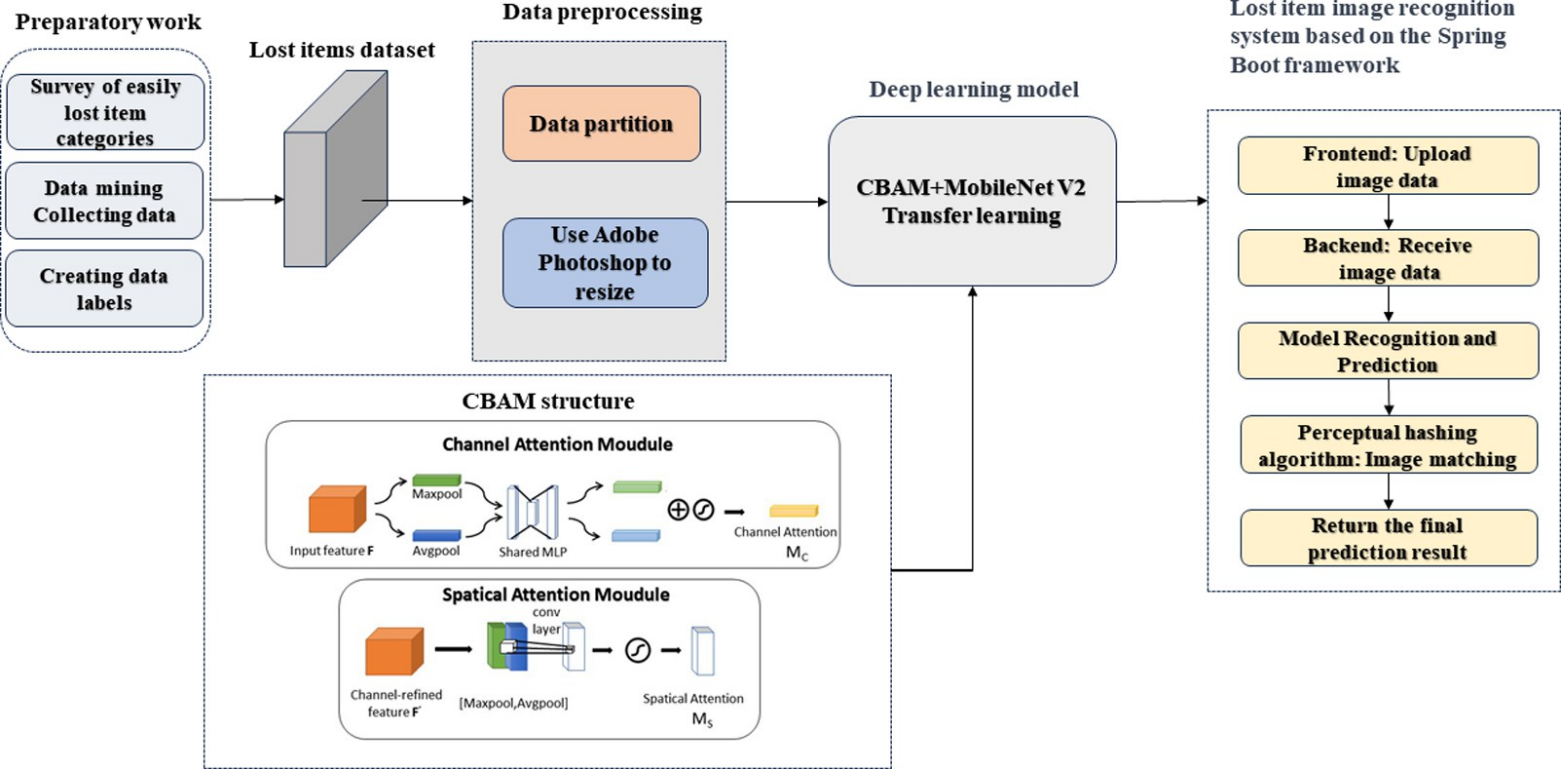
The overview of LostNet.

LostNet is constructed upon the MobileNet V2 architecture and incorporates the Perceptual Hash Algorithm and the CBAM to enhance perceptual capabilities and classification accuracy, as illustrated in Fig 1. To optimize the strengths of each component comprehensively, we have adopted a combination of two programming languages: Python and Java. This multilingual integration approach finds broad application across diverse domains, particularly in the development of large-scale application systems. Python excels in deep learning, scientific computing, and data processing, while Java’s strengths lie in enterprise applications, large-scale systems, and performance optimization. Key contributions of this study encompass:

- **First Lost and Found Dataset:** A novel dataset specifically designed for the lost and found domain has been introduced, created from diverse sources including web crawlers, real-world photography, and structured surveys.
- **Innovative Frontend-Backend Separation in Lost and Found Domain:** The LostNet algorithmic framework has been developed, integrating MobileNet V2, the CBAM attention mechanism, and the Perceptual Hash Algorithm. This integration demonstrates exceptional performance in the classification of lost item images.
- **High Efficiency and Accuracy:** The implementation achieves a testing accuracy of 96.8%, utilizing only 0.67 GFLOPs and 3.5M training parameters. This efficiency allows the system to recognize images in real-world scenarios and operate on a standard laptop, ensuring both high efficiency and low computational cost.

In this paper, we provide a detailed architectural overview of LostNet, the integration of the CBAM attention mechanism and the Perceptual Hash Algorithm, along with accompanying experimental results, as shown in Fig 1. By amalgamating Python and Java, our aim is to offer a potent tool for the field of lost item recognition, contributing to enhanced solutions for addressing lost item issues and improving the efficiency of social services.

The workflow comprises two main components: the frontend and the backend, coupled with the application of deep learning models and perceptual hash algorithms. In the frontend segment, users upload lost item images via a web interface, facilitated by the Layui framework, which ensures a user-friendly experience. In the backend section, built upon the Spring Boot framework, the system receives user-uploaded image data, subjecting it to multi-tier processing. Initially, images are forwarded to a deep learning model for online recognition, utilizing Python technologies, including Flask and PyTorch. The deep learning model employed is an enhanced MobileNet V2 model with the CBAM attention mechanism, yielding image classification results. Subsequently, uploaded images undergo processing with perceptual hash algorithms for image matching. The perceptual hash algorithm computes hash values or features, enabling comparisons with images stored in the database and achieving image matching. The backend then furnishes classification results from the deep learning model and potential matches to the frontend, enabling users to access recognition information and details of matched lost item images. The workflow harnesses the collaborative strengths of both frontend and backend components, drawing from diverse interdisciplinary fields, culminating in a robust system for lost item identification and matching. The design and implementation of this process draw upon expertise from fields such as computer vision and deep learning, ensuring efficient handling and recognition of lost item images.

### 3.1. MobileNet V2

An exemplary lightweight convolutional neural network is exemplified by MobileNetV2 [25]. This network incorporates reverse residuals and linear bottlenecks. The utilization of linear activation in the final layer of the inverted residual structure mitigates the risk of information loss. Furthermore, traditional convolutions are replaced with depth-separable convolutions, resulting in significant reductions in both computational load and model parameter count. Convolution on a depth-wise and point-wise scale are the two components that make up depth-separable convolution.


$${\text{O}}_{\text{x},\text{y},\text{c}}=\sum_{\text{w},\text{h}}^{\text{W},\text{H}}{\text{K}}_{\text{w},\text{h},\text{c}}\cdot {\text{I}}_{\text{x}+\text{w},\text{y}+\text{h},\text{c}}$$ (1)

In formula 1, the variable O represents the output feature graph, c represents the channel of the feature graph, x and y represents the coordinates on the output feature graph in the channel, and K epresents the convolutional kernel with a width W and height H, I represents the input feature graph, and w and h epresents the coordinates of the convolutional kernel weight element within the channel.

The primary distinction between point-by-point and standard convolution lies in the size of the convolution kernel. In point-by-point convolution, the kernel size is fixed at 1x1. The depth separable convolution process begins by utilizing depth convolution to extract the characteristics of each channel. Subsequently, point-by-point convolution is employed to correlate the extracted channel characteristics. The outcomes of this process are as follows:

$$\frac{{\text{R}}_{1}}{{\text{R}}_{2}}=\frac{{{\text{D}}_{\text{f}}}^{2}{{\text{D}}_{\text{k}}}^{2}\text{I}+{{\text{D}}_{\text{f}}}^{2}\text{IO}}{{{\text{D}}_{\text{f}}}^{2}{{\text{D}}_{\text{k}}}^{2}\text{IO}}=\frac{1}{\text{N}}+\frac{1}{{{\text{D}}_{\text{k}}}^{2}}$$ (2)

In formula 2, R_1_ and R_2_ represent the calculations of depth separable convolution and standard convolution, respectively; D_f_ and D_k_ represent the height and width of the input feature matrix; I represent the depth of the input feature matrix; and O represents the depth of the output feature matrix.

During feature extraction, MobileNetV2 employs depth-separable convolutions with a 3x3 kernel size, resulting in computational costs that are only 1/9th of those associated with standard convolutions. Remarkably, this reduction in computational cost comes with minimal impact on accuracy, highlighting one of the model’s distinctive strengths.

**Fig 2:**
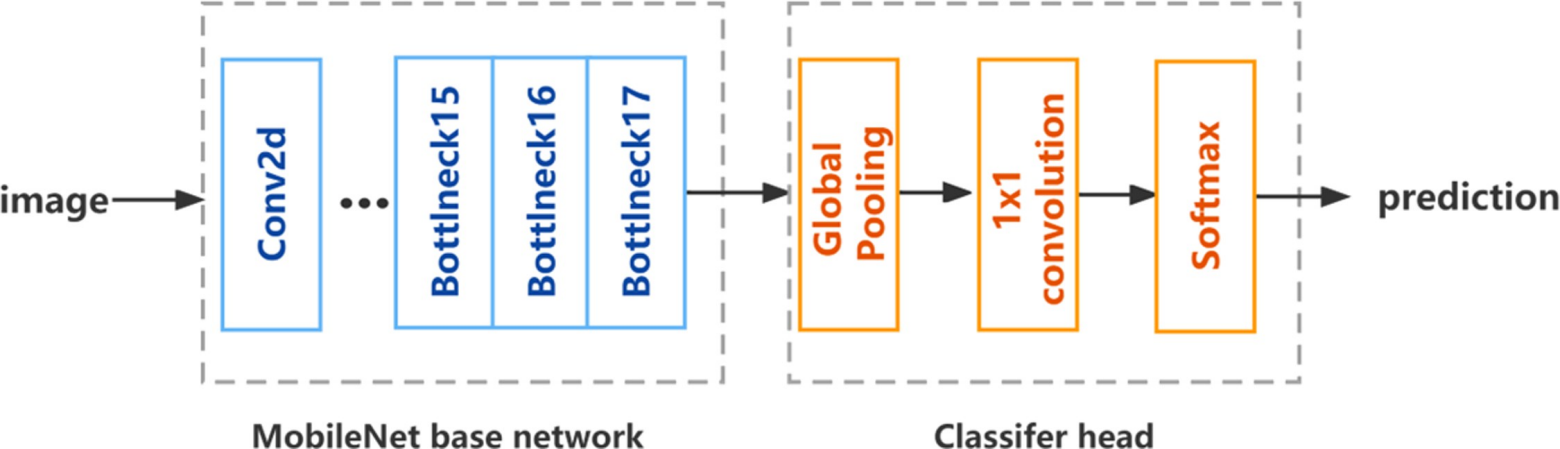
MobileNet V2 based classifier.

Fig 2 illustrates the organizational structure of the MobileNetV2 network, composed of three primary components. The front end consists of a CNN built with multiple convolutional layers. Subsequently, average pooling is applied to 1,280 blocks of size 7x7, resulting in the generation of 1,280 elements, which were then fully connected to one thousand neurons.

MobileNet V2 networks are ideal for deployment on mobile devices and embedded systems due to their compact model size, efficient processing capabilities, and rapid computation speed when compared to traditional convolutional neural networks. Furthermore, they meet the stringent speed requirements of CPUs.

### 3.2. Transfer learning

In straightforward terms, transfer learning involves leveraging existing knowledge to facilitate the acquisition of new knowledge [28]. Training a deep neural network model entirely from scratch for each task is a time-consuming and resource-intensive process. Such an approach encompasses laborious stages, including network initialization and feature extraction. In contrast, transfer learning optimizes the utilization of prior knowledge to address current challenges more efficiently. In the realm of deep learning, transfer learning involves incorporating pre-trained weights acquired from similar tasks. This strategy circumvents the need for training the model from the ground up, thereby reducing the computational cost associated with new learning. It expedites network convergence and enhances model stability and generalization capacity [29].

**Fig 3:**
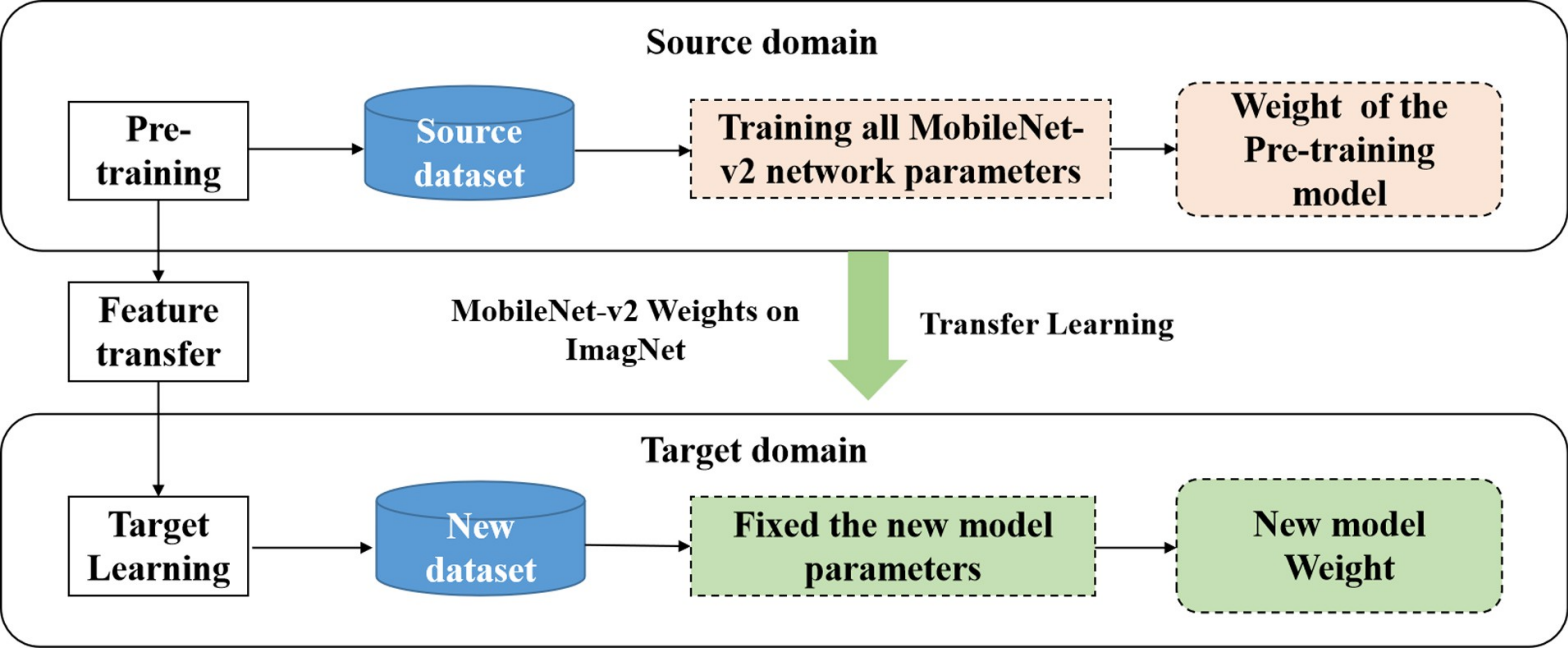
Transfer learning.

We initialized our model using pre-trained MobileNet V2 weights and fine-tuned it for our lost item classification task. Instead of downloading the entire ImageNet dataset, we utilized open-source MobileNet V2 weights. This transfer learning process is summarized in Fig 3. These pre-trained weights from ImageNet [30–32] encompass diverse image features, providing a robust foundation for our specific task. Our custom classifier includes a Dropout layer for overfitting prevention and a Dense Layer for category classification.

### 3.3. Convolutional block attention module

**Fig 4:**
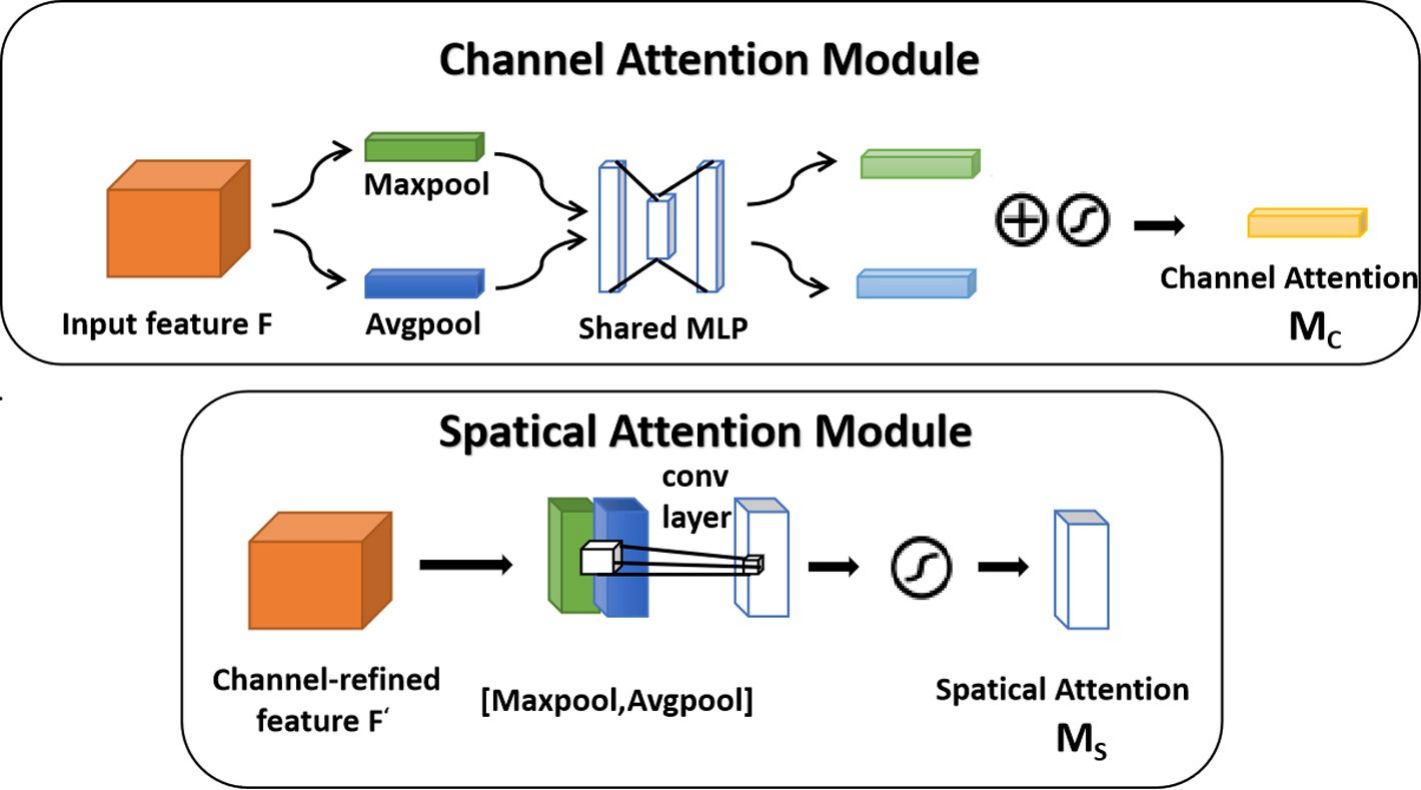
Channel attention module and spatial attention module.

The Convolutional Block Attention Module, often referred to as CBAM, is an attention mechanism module that combines both Channel and Spatial attentiveness. Its schematic diagram is illustrated in Fig 4. This module can be seamlessly integrated into the Model module, allowing for end-to-end training with the Model and adding only a negligible amount of additional processing [26].The Channel Attention Module takes an Intermediate Feature Map as input and produces both a 1-D Channel Attention Map and a 2-D Spatial Attention Map using CBAM. To aggregate spatial information, it employs both average pooling and maximum pooling methods, generating two descriptors. These descriptors are then forwarded to the same shared network, resulting in the production of the channel attention map. The complete channel attention map is acquired through the following steps:

$${\text{M}}_{\text{c}}\left(\text{F}\right)=\sigma (\text{M(a(F))+M(m(F))})=\sigma ({\text{W}}_{1}({\text{W}}_{0}({\text{F}}_{\text{avg}}))+{\text{W}}_{1}({\text{W}}_{0}\left({\text{F}}_{\text{max}}\right)))$$ (3)

The spatial attention module can highlight the information region and generate two 2D maps, which are then linked and convolved by a standard convolutional layer to produce a 2D spatial attention map. This map is produced after first averaging and maximum pooling along the channel to generate efficient feature descriptors. In conclusion, the formulation for the output of the spatial attention mapping is as follows:

$${\text{M}}_{\text{s}}(\text{F})={\sigma (\text{f}(\text{f}}_{\text{c}}{(\text{F}}_{\text{avg}},{\text{F}}_{\text{max}})))$$ (4)

In formulas 3 and 4, F is the input feature graph, is the sig-moid nonlinear activation function, M is the forward calculation function of multi-layer perceptron without bias, a and m are the mean and maximum pooling functions respectively, W_0_ and W_1_ are the weights of two linear layers, F_avg_ and F_max_ are the mean and maximum pooling functions respectively, σ represents the sig-moid nonlinear.

Sequentially arranging the Channel attention module and the Spatial attention module is essential to create CBAM. This empowers the model to focus on crucial features in both the channel and spatial dimensions while suppressing non-essential ones. The following offers a concise summary of the entire computation process:

$$\text{F}’={\text{M}}_{\text{c}}(\text{F})⊗\text{F}$$ (5)

$$\text{F}”={\text{M}}_{\text{s}}(\text{F}\prime )⊗\text{F}$$ (6)

In formulas 5 and 6, It represents the multiplication between the elements, and F′ is the weighted result of F passing through the channel attention mechanism, and F′′ is the weighted result of F′ passing through the spatial attention mechanism.

**Fig 5:**
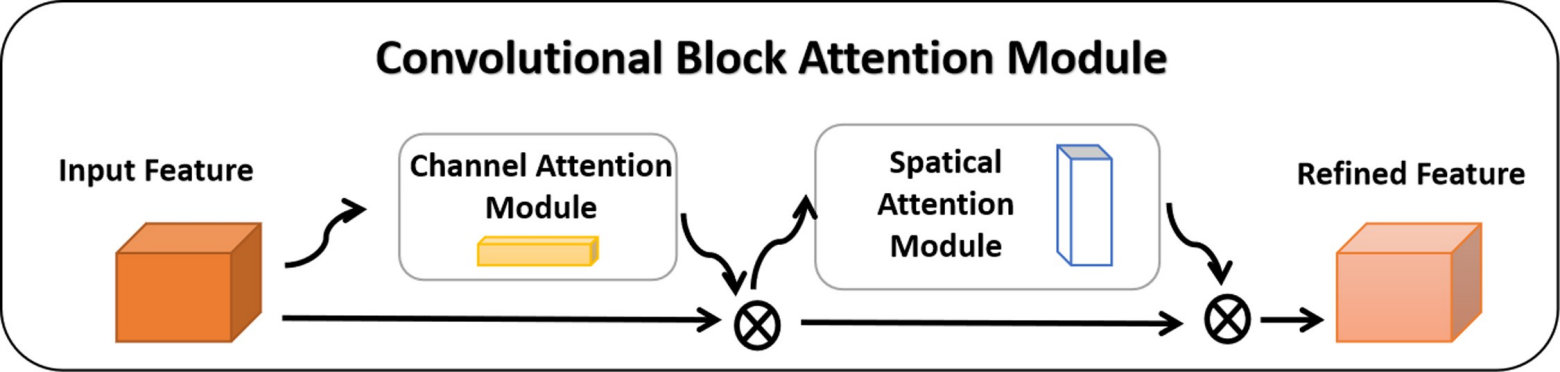
The overview of CBAM.

This algorithm incorporates an attention mechanism in both the channel and spatial dimensions, as shown in Fig 5. It enhances important features while suppressing less critical ones, thereby improving the model’s classification accuracy by emphasizing necessary features. The CBAM structure is introduced into the initial layer of the MobileNet V2 network through this algorithm. As CBAM is a lightweight module, it does not significantly impact the total number of model parameters, yet it enhances the focus on the model’s essential features. This ensures that the advantages of a lightweight model are effectively realized.

### 3.4. Perceptual hash algorithm

The perceptual hash algorithm begins by reducing the size of the picture and simplifying the colors [27]. Next, it aggregates the decomposition frequency and trapezoidal shape of the picture by using the discrete cosine transformation (DCT) method. After that, it reduces the DCT, keeps the 8*8 matrix in the upper right corner, calculates the average value of all 64 values, further reduces the DCT, and finally calculates the hash value. The algorithm creates a unique string that acts as a "fingerprint" for each image, and it then analyzes the results by contrasting the various "fingerprint" strings [33]. When the result is closer, it indicates that the picture is more comparable.

The advantage of using this approach is that the hash value remains similar even if the image undergoes modest modifications such as changes in height, width, brightness, or color. This robustness allows the algorithm to prevent the impact that would be caused by slight modifications of the image, ensuring consistent and reliable image matching.

An online image identification system is constructed in this research using the perceptual hash method and the concept of searching for images using photos.

### 3.5. Measurement

There is currently no standardized dedicated data collection that can be used for the categorization of photos of lost and found items. The study of confidential data sets is the focus of this work. Using the results of the poll, streamline the complicated lost and found categories into the top 10 categories that are the easiest to lose track of, with a total of 10499 photographs, as shown in Table 1. To ensure practical applicability, we avoided algorithmic pre-processing of the dataset. Directly scaling the collected lost and found dataset pictures to the input size of the network model will directly lead to the loss of some information in the picture, or even direct distortion, and will ultimately directly affect the image classification effect and recognition accuracy.This was due to the varying quality and high resolution of the original images. Therefore, prior to pre-training, we batch-processed the dataset using Adobe Photoshop for image standardization to 300 x 300 pixels. This resizing aimed at reducing overall file size for efficient data handling. This image processing step was applied to enhance suitability for training, distinct from algorithmic pre-processing, With the goal of improving sample data management and enhancing the ability of the model to generalize its findings.The 10,499 common lost and lost photographs augmented by the data were divided into the training set with 70% and the validation set with 30%.

**Table 1:** Dataset-specific category data.

Class name	Number of images
**bag**	1036
**book**	1067
**card**	1043
**earphone**	1025
**key**	1070
**lipstick**	1066
**Phone**	1068
**umbrella**	1045
**USB flash disk**	1020
**vacuum CPU**	1059

This article employs Accuracy, Average Precision (AP), Recall, Precision, and Loss as model performance evaluation indicators to analyze the advantages and disadvantages of the model.

Calculating values using typical complex matrix approaches like accuracy, recall, and precision may be difficult. When it comes to image classification, the confusion matrix is most commonly employed to make a comparison between the classification and the actual measurement value. This allows for a more understandable and accurate description of the correctness of model categorization. The formula for the computation is as follows:

$$\text{Accuracy}=\frac{\text{TN}+\text{TP}}{\text{TN}+\text{FN}+\text{FP}+\text{TP}}$$ (7)

$$\text{Recall}=\frac{\text{TP}}{\text{TP}+\text{FN}}$$ (8)

$$\text{Precision}=\frac{\text{TP}}{\text{TP}+\text{FP}}$$ (9)

The number of successfully predicted positive samples is denoted by the letter TP in the formula7-9, and the number of correctly anticipated negative samples is denoted by the letter TN. FN is for the number of samples that had errors predicted for them when they were positive, and FP stands for the number of samples that had errors predicted for them when they were negative.

The performance of the model recognition accuracy of the selected model may be evaluated objectively by using the average accuracy rate. The mathematical formula for the calculation is as follows:

$$\text{AP}=\frac{\sum_{1}^{E}\text{Accuracy}}{\text{E}}$$ (10)

The term AP, which is utilized in the aforementioned formula 10, stands for the average accuracy of the model that was picked, while E stands for the total number of iterations, and Accuracy stands for the accuracy of each iteration of the model that was selected.

The degree to which the model’s predicted value and the actual value deviate from one another may be estimated using the loss value. If the loss value is lower, then the model’s predicted result will be closer to the actual outcome. The formulae for the calculations are as follows:

$$\text{LOSS}=\frac{1}{\text{N}}\sum_{\text{i}}{\text{L}}_{\text{i}}=\frac{1}{\text{N}}\sum_{\text{i}}-\sum_{\text{c}=1}^{K}{\text{y}}_{\text{ic}}\;{\text{log}(\text{p}}_{\text{ic}})$$ (11)

In the previous Eq 11, LOSS is a numerical representation of the loss value associated with the model that was chosen, and K is the total number of categories. P_ic_ is the anticipated probability that sample i belongs to category c. If the category is the same as the category of sample i, it has a value of 1. Otherwise, it has a value of 0. The cross-entropy loss function is convex, and it is possible to calculate the value that is best for the whole world.

## 4. Experimental results

### 4.1. Training

**Fig 6:**
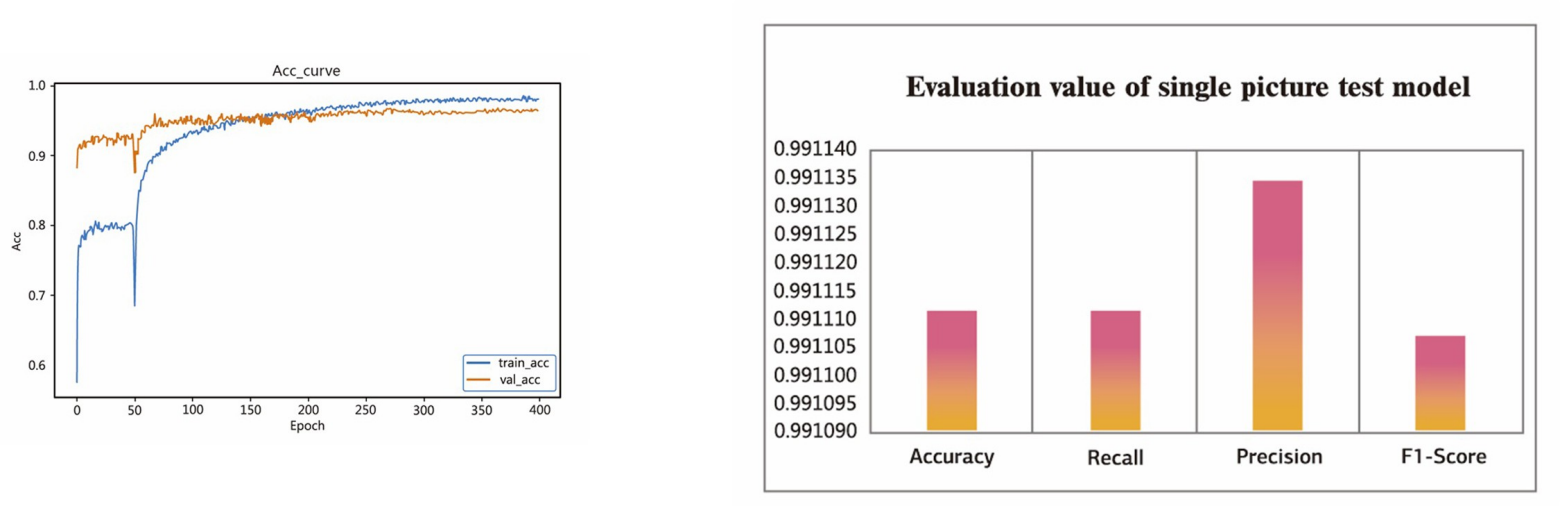
(a) Model Training and Validation Curve. (b) Evaluation Metrics Comparison.

In the course of our research, we looked at the problem of lost-object picture categorization. To classify images, we made use of the CBAM in conjunction with the well-known framework MobileNet V2. We utilized this architecture (transfer learning) to speed up the learning process while also reducing the amount of time needed for training. In order to evaluate the effectiveness of transfer deep learning, we examined the outcomes of three different optimizer functions: the Adaptive Moment Estimation Algorithm (ADAM), the Root Mean Square Propagation (RMSprop), and the Stochastic gradient descent (SGD) algorithm. We noticed that the optimizer SGD function functioned in a better method and got an accuracy of 96.8 percent, while the loss detected was 0.0047. We figured this out by looking at the Table 2. The results of the SGD function’s performance are presented in the form of a linear graph of the accuracy and single picture test evaluation in the **Fig 6**. SGD achieved notably shorter training times for the model compared to other optimizers, while all other model parameters remained optimal.

**Table 2:** Comparative chart of results using optimizer function.

Components	SGD	RMSprop	ADAM
**Accuracy/%**	96.8	89.5	94.7
**Loss**	0.0047	0.0050	0.0048
**Execution Time/ minutes**	5.38	14.28	10.71

It is abundantly clear, as evident from the preceding explanation accompanied by graphs and tables, that the SGD optimizer outperformed other optimization functions significantly when applied to the task of lost-object image classification. We conducted a comparative study involving nine transfer learning algorithm models, including EfficientNet [34], Inception V4 [35], ViT-B/32 [36], DenseNet201 [37], VGGNet19 [32], ShuffleNet V2 [38], ResNet152 [31], MobileNet V3 [39] and MobileNet V2 [25]. These models were selected randomly to assess the efficiency and effectiveness of the research approach presented in this study. The training outcomes are summarized in Table 3.

**Table 3:** Results of the selected model training.

Model	Accuracy/%	AP/%	Total parameters/M
**EfficientNet [34]**	93.9	89.0	66.348
**Inception V4 [35]**	89.5	80.3	41.158
**ViT-B/32 [36]**	94.9	94.3	104.766
**DenseNet201 [37]**	95.9	95.4	20.014
**VGGNet19 [32]**	95.6	94.7	139.611
**ShuffleNet V2 [38]**	88.6	82.3	2.279
**ResNet152 [31]**	87.8	81.9	60.193
**MobileNet V3 [39]**	95.7	94.9	5.483
**MobileNetV2 [25]**	95.4	94.6	3.505 (3.504872)
**LostNet**	96.8	96.2	3.505 (3.504991)

**Fig 7:**
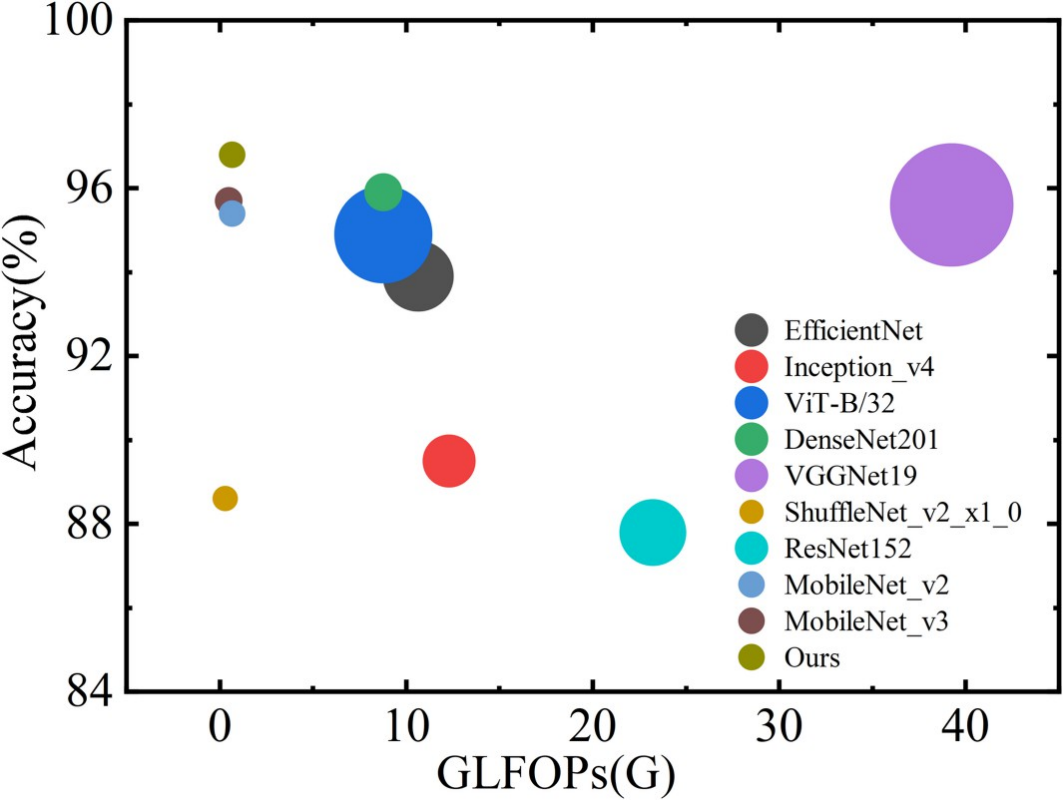
The performance, accuracy, and parameter amount compare of different models.

The results of the experiments demonstrate that the method under study has an average accuracy of 96.2% when applied to self-built data sets. This is a higher accuracy rate than EfficientNet, Inception V4, ViT-B32, DenseNet201, VGGNet19, ShuffleNet V2, ResNet152 and MobileNet V3 correspondingly. 7.2%, 15.9%, 1.9%,0.8%,1.5%, 13.9%, 14.3% and 1.3% of the total learning was transferred from other models. The suggested technique has obtained the greatest accuracy rate in private data sets, which is 96.8%, along with strong generalization ability and resilience. When compared to the similar kind of transfer learning model that has been developed, this is how it stands out. In correlation with the information shown in Fig 7, the ordinate in the figure shows the test accuracy, the abscissa represents GFLOPs, the circle colors reflect distinct transfer learning models, and the figure size denotes total parameters. However, due to the significant differences in the magnitude of total parameters among the models, a linear scaling method was employed to adjust the circle sizes. Specifically, the total parameters were scaled to a circle size range of 4.5 to 22 using the formula 12:

$$\text{Circle}\;\text{Size}=4.5+(22-4.5)\times \frac{{\text{param-min}}_{\text{param}}}{{\text{max}}_{\text{param}}{-\text{min}}_{\text{param}}}$$ (12)

where min_param_ and max_param_ are the minimum and maximum parameter values, respectively. This approach ensures that the visual representation of parameter sizes is balanced, making it easier to compare different models without overwhelming disparities. The suggested method achieves maximum accuracy on private datasets, demonstrating robustness and a strong ability to generalize.

When Fig 7 and Table 3 are considered together, a further complete evaluation of the performance of each model is carried out. This model is only second to MobileNet V3 and ShuffleNet V2, but it is worth recognizing that the Total parameters of the proposed model are almost half that of MobileNet V3 Total parameters, and the accuracy is higher than ShuffleNet V2 8.2%. MobileNet V3 and ShuffleNet V2 are the only models that are ahead of this model. In comprehensive comparison, the model that is being offered is a fantastic lightweight network, which provides the possibility that the model might be transplanted to mobile devices.

Importantly, as seen in Table 3, the addition of the CBAM attention mechanism to the initial layer of MobileNetV2 introduces a small number of additional parameters. Specifically, the Channel Attention Module and Spatial Attention Module within CBAM contain trainable parameters, such as the fully connected layers and convolutional layers. These additional parameters amount to approximately 0.000119M, which is minimal compared to the total parameter count of the model. Despite this slight increase, CBAM enhances the model’s representation capability by effectively weighting the input feature maps. This integration significantly improves feature recognition within our practical lost and found dataset while maintaining a relatively stable parameter size, which is beneficial for our subsequent platform development.

**Fig 8:**
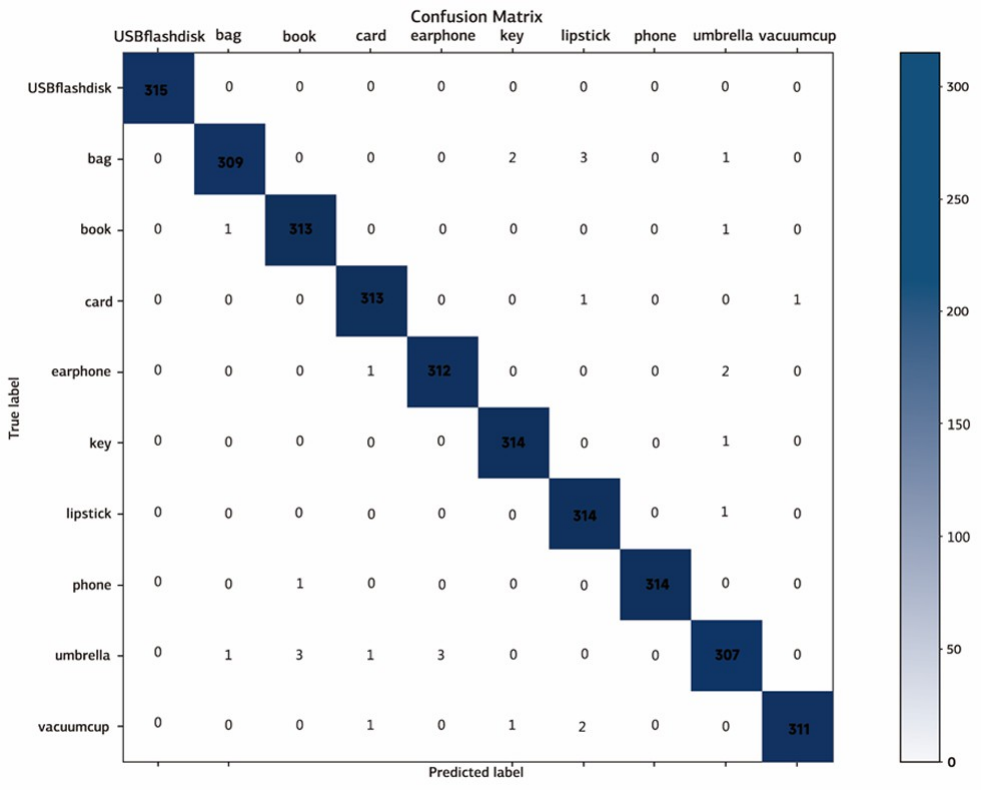
Confusion matrix of the model.

We decided to combine the confusion matrix from the model single-picture test with the ROC curve for the study so that we could have a more in-depth look at the data. The confusion matrix is the method for measuring the accuracy of classification models that is the most straightforward. Calculating the confusion matrix of the suggested model requires using Eqs (7) ~ (9). This matrix represents the identification result of the different types of lost object photographs and is derived from these equations. The confusion matrix is shown in Fig 8 with the rows representing the expected category for the item that was lost, and the columns representing the actual category for the object that was lost.

### 4.2. Inference

**Fig 9:**
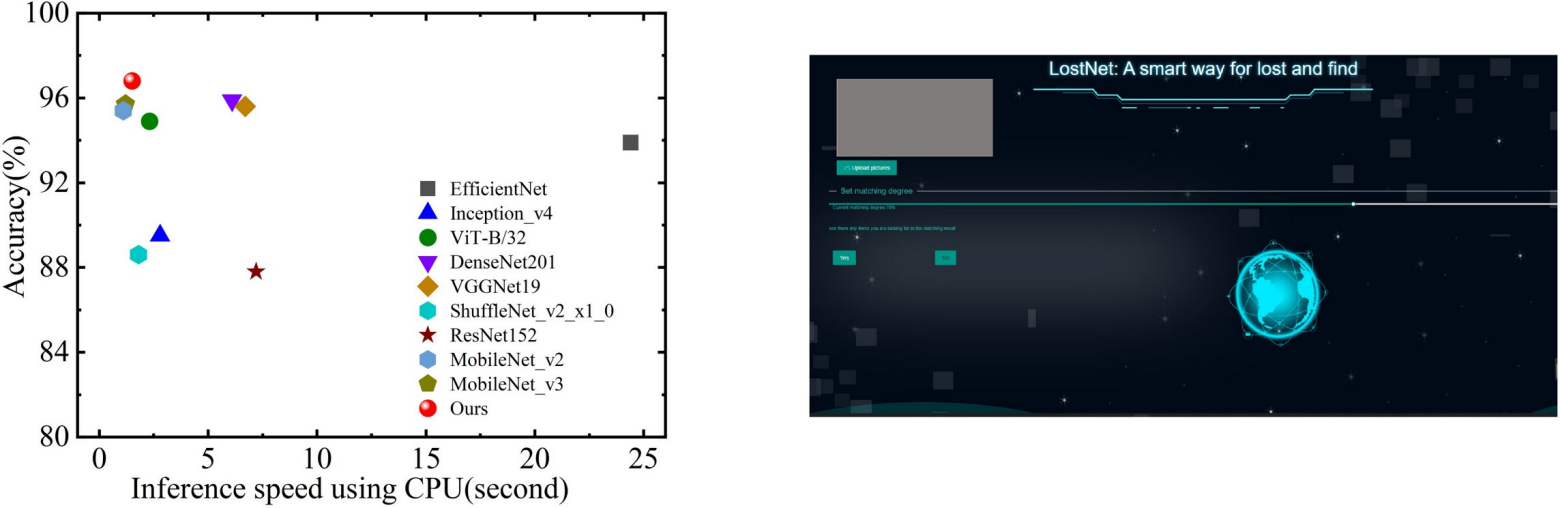
(a) CPU Inference Speed Comparison. (b)Real Engineering Application.

To successfully apply the model to the actual world, it needs to be simple enough that it can be executed on most laptops. In the cause of accomplishing this, we implement this model as well as a model that is very close to it on a regular computer that has a CPU, and we comprehensively consider the inference speed of the computer as the evaluation index. It takes the model 1.5 seconds to reason per picture on the CPU, which is significantly shorter than the other standard migration model of this type or even 1/16 of the EfficientNet’s processing time. This can be seen in Fig 9(A). Because it is such an exceptionally lightweight network, it is not hard to imagine that the concept will eventually be adapted to work with other mobile devices in the not-too-distant future. In light of this, we provide an engineering system that integrates the perceptual hashing method.

This paper, which is based on model training using the PyTorch and torchvision machine learning frameworks, uses the Spring Boot framework as the back-end service of the web page to realize the online recognition of pictures. The front-end web page utilizes the Layui framework for user-friendly web interface design and for facilitating the online Matching of user-uploaded images. The steps involved in the online identification procedure are as follows: the user launches the browser, navigates to the system’s website, navigates to the system’s home page, clicks the button to upload the local image, and the front end sends the POST request to the back end, and the image is transmitted to the back end. After the back end has received the picture that was uploaded by the user, it will first request the trained and upgraded MobileNet V2 migration learning model for prediction and recognition, and it will then return a category. After it has determined the category, it will next submit a request to the database, asking it to deliver an array containing the picture address associated with the category it has just determined. After obtaining the address, the image is then downloaded by using the image address, and then the perceptual hash algorithm is used to compare the downloaded image to the image that was uploaded by the user. The alignment will return a number for the similarity error, and the array will be used to send the few photos that have the least significant value for the similarity error to the front end. Fig 9(B) depicts the engineering interface after the model has been applied to the situation.

## 5. Conclusions

This research introduces a novel design approach aimed at addressing the challenges encountered in the management of urban transportation operations. Despite the widespread acclaim for merging convolutional neural networks with transfer learning in the field of image recognition, its application in the automatic recognition of lost and found remains limited. This study proposes a new technique for intelligent image identification based on a hash algorithm, leveraging its inherent advantages. The proposed approach, which adopts the concept of "search by map," is grounded in the utilization of a hash algorithm. To effectively handle the substantial number of missing images in the library and the abundance of extracted feature points, the CBAM structure is integrated into the network’s first layer. This integration introduces an attention mechanism in the dimensions of channel and space, thereby accentuating the requisite features and ultimately enhancing the model’s classification accuracy.

Moreover, an improved MobileNet V2 is employed to establish a transfer learning training model for identifying commonly lost objects. This facilitates a reduction in the number of comparisons between lost objects and database pictures, thus enabling more convenient object searches. Extensive experimental analyses conducted on photos sourced from the loss-object dataset, employing various transfer learning models, reveal that the proposed model achieves high recognition accuracy even under extreme conditions of GFLOPs and Total parameters. Furthermore, the features derived by this model outperform those extracted by alternative approaches in classification tasks.

To further enhance the precision of identifying missing object categories, the proposed model is being developed and implemented in the search sector. However, the current implementation supports only 10 kinds of objects, which is a limitation in practical use-cases where a broader range of objects may be encountered. Consequently, the subsequent stage of this research will involve expanding the model to include more object categories. This expansion will facilitate utilization in a broader range of search scenarios, thereby improving the overall effectiveness of the lost and found system.
