# Correction: LostNet: A smart way for lost and find

**DOI:** 10.1371/journal.pone.0314697

**Published:** 2024-11-26

**Authors:** The PLOS ONE Staff

There are errors in the author affiliations. The correct affiliations are as follows:

Meihua Zhou^1^, Ivan Fung^2^, Li Yang^1^, Nan Wan^1^, Keke Di^1^, Tingting Wang^1,3^

**1** School of Medical Information, Wannan Medical Collage, Wuhu City, China, **2** The University of Hong Kong, Hong Kong, China, **3** School of Physics and Electronic Information, Anhui Normal University, Wuhu, China.

The publisher apologizes for the error.
